# Nationwide registry‐based trial of risk‐stratified cervical screening

**DOI:** 10.1002/ijc.35142

**Published:** 2024-08-15

**Authors:** Laila Sara Arroyo Mühr, Jiangrong Wang, Sadaf S. Hassan, Emel Yilmaz, Miriam K. Elfström, Joakim Dillner

**Affiliations:** ^1^ Center for Cervical Cancer Elimination, Department of Clinical Science, Intervention and Technology (CLINTEC) Karolinska Institutet Stockholm Sweden; ^2^ Center for Cervical Cancer Elimination Karolinska University Hospital Huddinge Stockholm Sweden

**Keywords:** cancer risk, cervical cancer elimination, cervical screening, HPV, risk‐stratified screening

## Abstract

In well‐screened populations, most cervical cancers arise from small groups of women with inadequate screening. The present study aims to assess whether registry‐based cancer risk assessment could be used to increase screening intensity among high‐risk women. The National Cervical Screening Registry identified the 28,689 women residents in Sweden who had either no previous cervical screening or a screening history indicating high risk. We invited these women by SMS and/or physical letter to order a free human papillomavirus (HPV) self‐sampling kit. The Swedish national HPV reference laboratory performed extended HPV genotyping and referred high‐risk HPV‐positive women to their regional gynecologist. A total of 3691/28,689 (12.9%) women ordered a self‐sampling kit and 10.0% (2853/28,689) returned a sample for testing. Participation among women who had never attended screening was low, albeit improved. Up to 22.5% of women in other high‐risk groups attended. High‐risk HPV types were detected in 8.3% of samples. High‐risk HPV‐positive women (238/2853) were referred without further triaging and severe cervical precancer or cancer (HSIL+) in histopathology were detected in 36/158 (23%) of biopsied women. Repeat invitations gave modest additional participation. Nationwide contacting of women with high risk for cervical cancer with personal invitations to order HPV self‐sampling kits resulted in high yield of detected CIN2+. Further efforts to improve risk‐stratified screening strategies should be directed to improving (i) the precision of the risk‐stratification algorithm, (ii) the convenience for the women to participate and, (iii) ensuring that screen‐positive women are followed‐up.

## Graphical abstract

What's new?

In generally well‐screened populations, most cervical cancers arise from small groups of women with inadequate screening. This proof‐of‐concept study explored registry‐based cancer risk assessment for optimizing screening intensity among high‐risk women. Individual cancer risks differed by >1000 times and <1% of women had high cancer risks. The 28,689 women in Sweden who had either no previous cervical screening results or a screening history indicating high risk were offered self‐sampling for human papillomavirus testing. The additional screening yielded a high positive predictive value (23%) for cervical pre‐cancer (HSIL+) among biopsied women, suggesting successful targeting of high‐risk women.

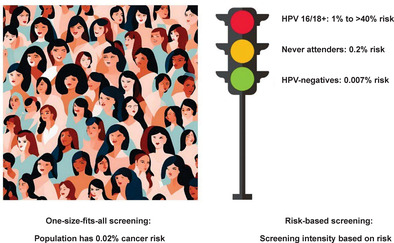

## 1 INTRODUCTION

Population‐based human papillomavirus (HPV) screening is a pillar of the global strategy for elimination of cervical cancer.^1^ Sweden has organized, population‐based cervical screening with >80% participation and only a few percent long‐term non‐attenders.^2^

The cervical cancer incidence in Sweden is currently >11/100,000 women‐years, but most women in the Swedish population always attend cervical screening when invited, have normal results and cancer risks <1/100,000 (data extracted from the annual report from the National Cervical Screening Registry, available online at https://nkcx.se/templates/_rsrapport_2022.pdf, accessed on February 16, 2024).

Most cervical cancers derive from a small group with either long‐term non‐attendance, inadequate screening and/or inadequate management after abnormal screening results.^3^, ^4^, ^5^ Cervical screening programs typically target entire populations (“one‐size‐fits‐all screening”) with no particular priority given to the small group of women with high cancer risks. The concept of “risk‐stratified screening” aims to improve efficiency and resource utilization by tailoring the screening intensity to the needs of each woman. The risk determinants present in a screening registry are (i) age, (ii) type of HPV detected (iii) long‐term non‐attendance, and (iv) cytological abnormalities, with atypical glandular cells (AGC) and other abnormalities detected after age 50 particularly associated with risk.^6^, ^7^ When all screening records are registered,^2^ it is straightforward to calculate the cervical cancer risk of each woman in the country.^3^, ^4^, ^5^, ^8^ We completed a pilot of reaching the highest risk women in 2019 using a combination of methods to send personal invitations.^8^ We have now continued with a nationwide implementation trial of risk‐stratified screening and report results of the enrolment, HPV status, and the corresponding follow up for participating women with baseline results from the trial in 2020–2021 and follow‐up of all women enrolled in 2019–2021.

## 2 MATERIALS AND METHODS

The trial is an adaptative trial that improves the risk assessment algorithm each year using current registry data, while also learning from the results of the outreach strategies used in previous years.

For all years, vaginal self‐sampling for HPV was offered to women at high risk for cervical cancer. For reasons of cost and logistics, self‐sampling kits were not sent to their home addresses, but women had to actively order them (opt‐in). All women received either a short message service (SMS) or a letter with an invitation to participate in the study.^8^ The invitation included a link for easy ordering of kits after providing an electronic informed consent.

### 2.1 Risk stratification

All cervical cytologies, histopathologies, HPV tests and invitations to screening in Sweden are registered in the National Cervical Screening Registry (NKCx.se).^2^ The registry was followed up for risk of subsequent invasive cancer after the different screening test results. Data from the NKCx linkage was used for estimation of the five‐year cumulative risk, as presented in Table 1. When data was not available, data from the literature was used (detailed in Supplementary Table S1). Age‐specific risks were estimated in proportion to the age‐specific cervical cancer mortality rates in Sweden (Supplementary Table S1).

**TABLE 1:** Five‐year cumulative risk of invasive cervical cancer (in percent) based on age, result of HPV test and result of cytology.

Cytology	HPV status	All ages	By age group									
			23–29	30–34	35–39	40–44	45–49	50–54	55–59	60–64	65–70	71–75
AGC in last two screening intervals	16/18+	18.3	4.5	9.6	10.5	18.3	16.7	22.8	23.6	24	32.9	43.8
	Other HPV+	4.6	1.1	2.4	2.6	4.6	4.2	5.7	5.9	6	8.2	10.9
	No HPV test	2.1	0.5	1.1	1.2	2.1	1.9	2.6	2.7	2.7	3.8	5
HSIL in last two screening intervals	16/18+	10	2.5	5.2	5.7	10	9.1	12.5	12.9	13.1	18	23.9
	Other HPV+	3.3	0.8	1.7	1.9	3.3	3	4.2	4.3	4.4	6	8
	No HPV test	2.9	0.7	1.5	1.7	2.9	2.6	3.6	3.7	3.8	5.2	6.9
ASCUS/LSIL in last two screening intervals	16/18+	1.1	0.3	0.6	0.6	1.1	1	1.3	1.4	1.4	1.9	2.6
	Other HPV+	0·3	0.1	0.1	0.2	0.3	0.2	0.3	0.3	0.4	0.5	0.6
	No HPV test	0·4	0.1	0.2	0.2	0.4	0.4	0.5	0.5	0.5	0.7	1
HPV16/18+, normal cytology in last two screening intervals		1.3	0.3	0.7	0.7	1.3	1.2	1.6	1.7	1.7	2.3	3.1
Never attended in 20 years		0.2	0.05	0.1	0.11	0.2	0.18	0.25	0.26	0.26	0.36	0.48
High‐risk HPV negative in last two screening intervals		0.0074	0.0018	0.0039	0.0042	0.0074	0.0067	0.0092	0.0096	0.0097	0.0133	0.0177
Total population		0.02	0.04	0.04	0.07	0.07	0.09	0.09	0.09	0.02	0.13	0.17

*Note*: Risks by age group were estimated in proportion of age‐specific cervical cancer mortality rates in Sweden. Details of data source and publications used to estimate the risks were presented in Supplementary Table S1.

Abbreviations: AGC, atypical glandular cells; ASCUS, atypical squamous cells of undetermined significance; HSIL, high‐grade squamous intraepithelial lesion; LSIL, low‐grade squamous intraepithelial lesion.

### 2.2 Study population

**FIGURE 1:**
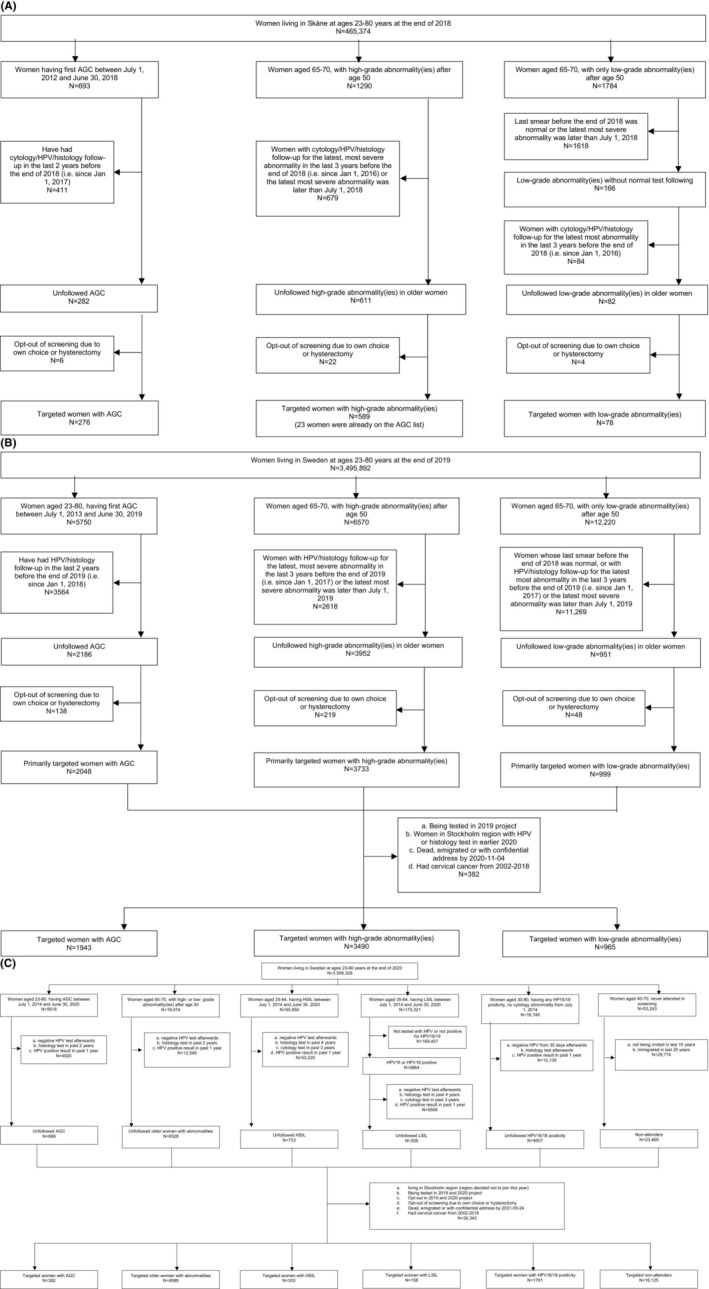
Flow‐chart on identification of high‐risk women performed in (A) 2019 trial (adapted from Wang et al.^8^), (B) 2020 and (C) 2021 trial. AGC, atypical glandular cells; HSIL, high‐grade squamous intraepithelial lesion; LSIL, low‐grade squamous intraepithelial lesion.

The 2019 pilot was restricted to one county in Sweden and only two high risk groups: (i) AGC in screening tests during the past 0.5–6.5 years (the timeframe corresponding to two screening intervals; buffer period of 0.5 years is to avoid inviting women who might be under active follow‐up) and (ii) abnormal screening results above the age of 50, but without sufficient follow‐up. There were 920 women with these profiles in the county who were invited (Figure 1A).

The study was extended to the whole of Sweden in 2020. The AGC group was defined as above, but this year also required no HPV test or histopathology performed in the previous 2 years (to avoid inviting women who were under active follow‐up). The group of elderly women with inadequate follow‐up of abnormal cytology after 50 was defined as women ages between 65 and 70, with either a high‐grade abnormality after age 50, with no HPV or histopathology test in the previous 3 years (registry‐based estimate of duration of cancer protection following those tests) or with a low‐grade abnormality after age 50, with no normal cytology afterwards and no HPV or histopathology test in the previous 3 years (Figure 1B).

For 2021, the trial included the whole of Sweden except for the Stockholm region (this region switched to primary screening with HPV self‐sampling during 2021) and this year the trial also targeted never‐attenders (defined as women ages 40–70, with no cervical tests at all in the national screening registry NKCx [that is complete since 1995]), that had been invited to screening during the last 10 years, and had been resident in Sweden for at least 20 years. The AGC group was identified as in 2020 plus that there were no negative HPV test afterwards and no HPV positive tests since January 1, 2020 (HPV negativity reduces risk and recent HPV positivity implies ongoing follow‐up). The elderly group were women aged 65–70 years, with a squamous intraepithelial lesion (SIL, including high grade [HSIL] and low grade [LSIL]) after age 50, without a negative HPV test afterwards, without a biopsy since January 1, 2019, and without an HPV positive test since January 1, 2020. Furthermore, another three risk groups were invited: (1) HSIL: Women ages 35–64, with HSIL in the past 0.5–6.5 years, without a negative HPV test afterwards, without a biopsy since January 1, 2017, without a cytology test since January 1, 2018, and without a HPV positive test since January 1, 2020, (2); 16/18 LSIL: Women ages 35–64, with LSIL in past 0.5–6.5 years (i.e., between July 1, 2014 and June 30, 2020) and HPV16/18 positive, without a negative HPV test afterwards, without a biopsy since January 1, 2017, without a cytology test since January 1, 2018, and without an HPV positive test since January 1, 2020; and finally (3) HPV16/18‐positive with normal cytology: women ages 30–80, with an HPV16 or HPV18 positive test since July 1, 2014, without a negative HPV test at least 30 days after the positive index sample, without a biopsy afterwards, and without an HPV positive test since January 1, 2020 (Figure 1C).

The trial targeted women at high risk of cervical cancer (regardless of whether guidelines had been followed or not). Major exclusion criteria were a cervical cancer diagnosis during 2002–2020 and having opted out from the screening program. The Total Population Register identified addresses and the additional exclusion criteria: death, emigration and confidential addresses. Women could also opt out by notifying the study at the website, by phone call, by mail and as a free OPT OUT reply SMS.

### 2.3 Invitations

The women were invited (either by an SMS or by physical letters [when the women did not have a mobile phone]) to order a free HPV self‐sampling kit via the study web‐platform (https://www.hpvcenter.se/kit/index.php). Women first provided informed consent and then ordered the self‐sampling kit for home delivery. Only the 28,689 women whose personal identity number (PNR) were in the project database could order a kit and only one kit per PNR.

In 2019, women were invited by SMS and received 2 more SMS reminders (1 and 2 months after initial invitation) if they had not ordered a self‐sampling kit. A physical letter was sent to all women who still had not ordered a self‐sampling kit after the three SMS and to all women who did not have a mobile phone.^8^

In 2020, women were invited by SMS and by physical letter (if no mobile phone) in a similar manner. SMS reminders were sent 15 and 21 days after the first SMS. Women who ordered a self‐sampling kit but did not send in the sample, were sent 2 SMS reminders. For women without a phone who had ordered a kit but not sent in the sample, a physical letter including a new self‐sampling kit was sent.

In 2021, women were similarly invited by SMS or by physical letter. SMS reminders were sent 15 days and 2 months after the first SMS. No reminders were sent for women who had ordered a self‐sampling kit and did not return it for HPV analysis (they were instead sent a new kit in 2022, if they still fulfilled the risk criteria).

Women who participated in the trial and had an HPV result resulting in that they no longer belonged to a risk group were not invited again in the next year. Women that did not order a test were included in the following year, if they still fulfilled the criteria for a high‐risk group.

### 2.4 HPV self‐sampling kit

The self‐sampling kits used were the same as the ones used by the cervical screening program. HPV testing platform was selected by a competitive purchasing tender, which was won by the Roche Cobas platform and included the sampling materials corresponding to the platform (The Cobas® PCR Female Swab Sample Packet).

### 2.5 HPV analyses

The tendered HPV platform (Cobas® 4800) tests for HPV16, HPV18, or “other” HPV. Samples classified as “other,” were HPV genotyped using MGP5+/6+ general PCR followed by Luminex HPV genotyping, as described.^9^ Individual HPV types are in this report classified as the highest oncogenicity viruses: HPV 16, HPV 18, HPV 45; medium oncogenicity (HPV types 31, 33, 52, 58) and low oncogenicity (35, 39, 51, 56, 59, 66, 68).

### 2.6 Follow up

HPV16, 18, or 45‐positive women were referred to the gynecologist responsible for diagnostic work‐up within the cervical screening program in her region of domicile. Results on all cytologies and histopathologies performed after the HPV test anywhere in Sweden (also if performed by someone else than the regionally responsible gynecologist) were retrieved from the Swedish National Cervical Screening Registry (NKCx.se).

Women with medium or low oncogenicity HPV types were informed about the results, but not referred to a gynecologist. However, they could book a time if concerned. Furthermore, all women positive for medium or low oncogenicity HPVs during 2019–2021, were sent a new self‐sampling kit (at least 1 year later). If the HPV result in the second sample was the same (HPV persistence), the woman was referred.

### 2.7 Evaluation and statistical analysis

The evaluation criteria (outcomes) were the participation (the number and proportion of kits ordered and returned), the proportion of HPV positivity among returned samples, and the positive predictive values for CIN2+ in biopsy‐confirmed histopathology among HPV‐positive participants. Binomial proportions were calculated with corresponding 95% exact confidence intervals using SAS 9.4, Cary NC, USA.

## 3 RESULTS

In the overall trial (women enrolled 2019 to 2021 and followed up to December 31, 2022) we invited 28,689 high risk women (Table 2). The major risk groups were never‐attending women (*n* = 16,125), women aged between 65 and 70 who had an HSIL/LSIL above age 50 with inadequate follow‐up (*n* = 8148), women with AGC (*n* = 2193), HPV16/18‐positive (but cytologically normal) women who had not been followed up (*n* = 1761), and women (aged 35–64) with HSIL or LSIL but inadequate follow‐up (*n* = 303 and 159, respectively) (Table 2).

**TABLE 2:** Study population, participation, and HPV positivity.

Recruitment	Number of women	Number who ordered	% ordered among women (95% CI)	Number of tested	(% tested among women)	Number of HPV +ve	HPV +ve among tested (%)	HPV 16	HPV 18	HPV 45	High oncogenicity (%, [95% CI])	Medium oncogenicity	Low oncogenicity	Negative
AGC^a^	2193	441	20.1 (18.5–21.9)	354	16.14	50	14.12	10	6	9	25 (7.1 (4.6–10.3)	11	14	304
Elderly^b^	8148	2015	24.7 (23.8–25.7)	1831	22.47	228	12.45	60	14	33	107 (5.8 (4.8–7.0)	54	67	1603
HSIL^c^	303	37	12.2 (8.8–16.4)	23	7.59	4	17.39	2	0	0	2 (8.7 (1.1–2.8)	1	1	19
16/18 LSIL^d^	159	25	15.7 (10.4–22.3)	16	10.06	5	31.25	2	1	0	3 (18.75 (4.1–45.7)	2	0	11
HPV16/18^e^	1761	297	16.9 (15.1–18.7)	193	10.96	107	55.44	71	16	5	92 (47.7 (40.5–55.0)	6	9	86
Never attenders^f^	16,125	876	5.4 (5.1–5.8)	436	2.70	23	5.28	7	1	1	9 (2.1 (1.0–3.9)	8	6	413
Total	28,689	3691	12.9 (12.5–13.4)	2853	9.94	417	14.62	152	38	48	238 (8.3 (7.4–9.4)	82	97	2436

^a^ Women aged between 23 and 80, having atypical glandular cells (AGCs) in past 0.5–6.5 years and no HPV or histopathology test in the last 2 years.

^b^ Women aged between 65 and 70 with abnormal screening findings above the age of 50, but without sufficient follow‐up (risk >65/100,000).

^c^ Women aged 35–64, having high‐grade squamous intraepithelial lesions (HSIL) in past 0.5–6.5 years, not having any negative HPV test afterwards, not having histology test since January 1, 2017, not having cytology test since January 1, 2018, and not having HPV positive test since January 1, 2020.

^d^ Women aged 35–64, having low grade squamous intraepithelial lesions (LSIL) in past 0.5–6.5 years (i.e., between July 1, 2014 and June 30, 2020) and tested HPV16 or HPV18 positive, not having any negative HPV test afterwards, not having histology test since January 1, 2017, not having cytology test since January 1, 2018, and not having HPV positive test since January 1, 2020.

^e^ Women aged 30–80, ever having HPV16 or HPV18 positive and no cytology abnormality since July 1, 2014, not having any negative HPV test from 30 days after the positivity, not having any histology test afterwards, and not having HPV positive test since January 1, 2020.

^f^ Women aged 40–70, never attended screening (no data on screening in the NKCx, which is nationwide complete since 1995), being invited in last 10 years, and not immigrated in last 20 years.

In total, 12.9% (3691/28,689) of women ordered a self‐sampling kit. The group of women aged between 65 and 70 with HSIL/LSIL had the highest participation (24.7%, 2105/8148). Only 5.4% (876/16,125) of the never‐attending women ordered a self‐sampling kit (Table 2).

Up to 77.3% (2853/3691) of the ordered kits were returned. More than 80% of women with AGC returned the sample, but only 49.8% of kits ordered by never‐attending women were returned (Table 2).

HPV was detected in 417 self‐samples (14.6%). Most positivities were among the women who had been HPV16/18 positive but not followed up 55.4% (107/193) were positive for the same type again, with 81.3% (87/107) having HPV16 and 18 (71 and 16 women, respectively). The never‐attenders had a low HPV prevalence (5.3%) (23/436) (Table 2).

Most HPV‐positive samples contained HPV16 (*n* = 152). There were 97 women with low oncogenicity HPV types (HPV35/39/51/56/59/66/68) (*n* = 97), 82 women with medium oncogenicity HPV types (HPV 31/33/52/58), and 48 women with HPV 45. HPV18 was detected in 38 women (Table 2).

This is an adaptive trial based on individual risks and the exact risk profile of the women in Sweden is changing every year when new screening information is recorded. The exact results obtained in each year are found in Supplemental Material (Supplementary Table S2). The sum of the total amount of women per year is higher than the overall 2019–2021 number, as some women were identified as high‐risk women in several consecutive years. When reporting the overall trial, each woman is only considered once.

### 3.1 Follow up of HPV‐positive women

HPV 16, 18, or 45‐positive women were referred to a regional gynecologist for diagnostic workup, including cytology and colposcopy‐directed biopsy. All cytological and histopathological results in Sweden are reported to the Swedish National Cervical Screening Registry (NKCx.se). Subsequent cytologies and histopathologies were retrieved for all consenting women, including from women who had not been referred (such as HPV‐negative women).

There were 417 HPV‐positive women out of which 238 were positive for HPV 16, 18, or 45 (Table 3). Only 158/238 had a cervical biopsy taken (Table 3). For 36/158 women (22.8%), an HSIL+ was found on histopathology. The positive predictive value (PPV) for HSIL+ was 25.0% among women who had HPV16/18‐positivity, despite having had normal cytology (Table 3). The few never‐attenders who were positive for HPV types 16/18/45 and followed up also had a high PPV for HSIL+ (Table 3).

**TABLE 3:** Results on histopathological examinations of cervical biopsies taken after enrollment.

	Histopathology										
	Total	Total histo	Normal	LSIL	CIN1	HSIL	AIS	Cancer	Not diagnostic	Other^g^	PPV CIN2+
AGC^a^											
HPV16	10	8	3	3	0	1	1	0	0	0	25.0
HPV18	6	4	0	0	0	1	3	0	0	0	100.0
HPV45	9	7	5	1	0	0	0	0	1	0	0.0
Other HPV medium oncogenicity	11	3	2	0	0	1	0	0	0	0	33.3
Other HPV low oncogenicity	14	6	5	1	0	0	0	0	0	0	0.0
Negative	304	11	8	3	0	0	0	0	0	0	0.0
Elderly^b^											
HPV16	60	34	19	7	2	5	0	1	0	0	17.7
HPV18	14	10	8	2	0	0	0	0	0	0	0.0
HPV45	33	17	14	3	0	0	0	0	0	0	0.0
Other HPV medium oncogenicity	54	14	6	5	0	2	0	0	1	0	14.3
Other HPV low oncogenicity	67	15	9	2	0	4	0	0	0	0	26.7
Negative	1603	24	17	4	0	0	0	0	2	1	0.0
HSIL^c^											
HPV16	2	2	0	0	0	2	0	0	0	0	100.0
HPV18	0	NA	NA	NA	NA	NA	NA	NA	NA	NA	NA
HPV45	0	NA	NA	NA	NA	NA	NA	NA	NA	NA	NA
Other HPV medium oncogenicity	1	0	0	0	0	0	0	0	0	0	NA
Other HPV low oncogenicity	1	0	0	0	0	0	0	0	0	0	NA
Negative	19	0	0	0	0	0	0	0	0	0	NA
LSIL^d^											
HPV16	2	2	2	0	0	0	0	0	0	0	0.0
HPV18	1	1	0	1	0	0	0	0	0	0	0.0
HPV45	0	NA	NA	NA	NA	NA	NA	NA	NA	NA	NA
Medium oncogenicity HPV	2	0	0	0	0	0	0	0	0	0	NA
Low oncogenicity HPV	0	NA	NA	NA	NA	NA	NA	NA	NA	NA	NA
Negative	11	0	0	0	0	0	0	0	0	0	NA
HPV16/18+/Cyt‐neg^e^											
HPV16	71	53	22	16	1	13	0	0	1	0	24.5
HPV18	16	13	6	3	0	0	1	3	0	0	30.8
HPV45	5	2	2	0	0	0	0	0	0	0	NA
Medium oncogenicity HPV	6	2	0	2	0	0	0	0	0	0	NA
Low oncogenicity HPV	9	2	0	1	0	0	0	0	1	0	NA
Negative	86	7	3	3	0	0	1	0	0	0	14.3
Never attenders^f^											
HPV16	7	4	0	0	0	4	0	0	0	0	100.0
HPV18	1	0	0	0	0	0	0	0	0	0	NA
HPV45	1	1	0	0	0	1	0	0	0	0	100.0
Other HPV medium oncogenicity	8	1	0	0	0	1	0	0	0	0	100.0
Other HPV low oncogenicity	6	1	0	1	0	0	0	0	0	0	0.00
Negative	413	5	2	2	0	0	0	0	1	0	0.00
Total HPV 16, 18, 45 positive	238	158	81	36	3	27	5	4	2	0	22.8 (16.5–29.3)
Total HPV positive	417	202	103	48	3	35	5	4	4	0	21.8 (16.3–28.1)

*Note*: All histopathologies in the entire country of Sweden and all enrolled women (also HPV‐negative women that were not referred are included in the linkage).

Abbreviations: AGC, atypical glandular cells; AIS, adenocarcinoma in situ; CIN1, cervical intraepithelial neoplasia grade 1; HSIL, high‐grade squamous intraepithelial lesion; LSIL, low‐grade squamous intraepithelial lesion.

^a^ Women aged between 23 and 80, having AGC in past 0.5–6.5 years and no HPV or histopathology test in the last 2 years.

^b^ Women aged between 65 and 70 with abnormal screening findings above the age of 50, but without sufficient follow‐up (risk >65/100,000).

^c^ Women aged 35–64, having HSIL in past 0.5–6.5 years, not having any negative HPV test afterwards, not having histology test since January 1, 2017, not having cytology test since January 1, 2018, and not having HPV positive test since January 1, 2020.

^d^ Women aged 35–64, having LSIL in past 0.5–6.5 years (i.e., between July 1, 2014 and June 30, 2020) and tested HPV16 or HPV18 positive, not having any negative HPV test afterwards, not having histology test since January 1, 2017, not having cytology test since January 1, 2018, and not having HPV positive test since January 1, 2020.

^e^ Women aged 30–80, ever having HPV16 or HPV18 positive and no cytology abnormality since July 1, 2014, not having any negative HPV test from 30 days after the positivity, not having any histology test afterwards, and not having HPV positive test since January 1, 2020.

^f^ Women aged 40–70, never attended screening, being invited in last 10 years, and not immigrated in last 20 years.

^g^ The “other diagnosis” was a vaginal cancer.

### 3.2 Re‐invitation of women in successive years

Women who did not participate in the study in one calendar year, could be invited again in the next calendar year if they still were at high risk. Of course, if a woman had e.g., attended routine screening during the year, she would no longer be a never‐attender and would not be eligible for invitation again within the trial.

There were 232/276 (84.1%) women with AGC and 525/644 (81.5%) elderly women who had been invited and not participated in 2019. Out of these, 127 women with AGC and 332 elderly women were invited again in 2020, and 5.5% (7/127) women with AGC and 6.6% (6/332) elderly women chose to participate in 2020. (Table 4). Similarly, there were 298 women with AGC and 1212 elderly women who had been invited and not participated in 2020 who were invited again in 2021. The participation after re‐invitation was similar after a first invitation, with 4.0% (12/298) women with AGC and 6.0% (73/1212) elderly women participating the following year (Table 4). A third invitation (second re‐invitation) did not result in any further increases in participation at all (Table 4). Women who had not participated after re‐invitation in 2020 were also invited in 2021 but none of them participated (0/140).

**TABLE 4:** Subsequent invitation and participation of prior non‐participants.

	Consecutive invitations for women first called in 2019	
	AGC	Elderly
Total invited	276	644
Participated	44	119
Not participated	232	525
Not invited again	105	193
Invited again	127	332
Participated	7	22
Not participated	120	310
Not invited again	107	193
Invited again	13	117
Participated	0	0
Not participated	13	117

	Consecutive invitations for women called in 2020	
	AGC	Elderly
Total invited	1943	4455
Participated	291	878
Not participated	1652	3577
Not invited again	1354	2365
Invited again	298	1212
Participated	12	73
Not participated	286	1139

*Note*: Atypical glandular cells (AGC): Women aged between 23 and 80, having atypical glandular cells in past 0.5–6.5 years and no HPV or histopathology test in the last 2 years. Elderly: Women aged between 65 and 70 with abnormal screening findings above the age of 50, but without sufficient follow‐up.

## 4 DISCUSSION

Our nationwide trial demonstrated registry‐based stratification of the population by cervical cancer risk differing >1000 times. Offering HPV‐screening to all women with high cervical cancer risk was feasible and resulted in a high positive predictive value among biopsied, screen‐positive women.

Strengths of the study include that both identification of risks and follow‐up for results was performed nationwide, including all women and laboratories in the country, and that it constitutes a first proof‐of‐concept of national risk‐stratified screening. Although there are no similar trials, self‐sampling for HPV has successfully been used in many trials to increase screening attendance.^10^ It was therefore not ethically possible to use randomization, but the trial uses the routine nationwide screening program (that is followed up using the same registry in exactly the same manner) as comparison for evaluation of the outcomes of the intervention.

Weaknesses of the trial include limited generalizabity, as a screening registry is required to estimate individual risks and some countries may not have that. For some subgroups in the population (e.g., HPV16/18‐positive AGC) the number of observations is limited and risk estimations in small age bands may be uncertain. It should be noted that some of the risk estimates may appear counterintuitive (e.g., HSIL having lower risk than AGC and ASCUS having lower risk than normal cytology). The trial has estimated the real‐life risks after the findings and if women are adequately followed‐up and treated, the risks are of course lower. For example, HSIL is regularly followed‐up/treated whereas follow‐up of AGC may be more irregular. Also, HPV‐positive ASCUS is directly referred for management, whereas HPV‐positive women with normal cytology are typically only followed.

The participation rate in our trial (12.9%) was comparable to most trials using self‐sampling to target long‐term non‐attending women.^10^ Most women in the trial (56.2%) were never‐attenders. In the routine program, non‐attenders are issued a new screening summons every year. The low participation in this trial (5.4%) is actually much better than the participation obtained by the annual repeat screening summonses.^10^

The high PPV among referred women, in spite of no triaging, is not surprising, as long‐term follow‐up of persistently HPV‐positive women have found very high rates of progression to HSIL+.^11^ The high PPV implies that when targeting high‐risk women, triaging among HPV‐positives is not needed which could simplify management and minimize attrition.

Previous studies have found that the participation rate is improved about twice as much if self‐sampling kits are sent directly to the home of the women, without having to order the kit first,^10^ which could be an important improvement for the present study. Furthermore, the use of extended HPV genotyping in the routine program could be used to improve the risk stratification as different oncogenic HPV types are known to differ in their cancer risk by about 50 times.^12^ Finally, efforts should be directed towards ensuring that follow‐up of HPV‐positive women does happen.

In conclusion, risk‐stratified screening is a promising concept for using updated registry information on how to tailor a screening program for improved efficiency. The current study has provided proof of concept showing that it also works in practice.

## AUTHOR CONTRIBUTIONS

**Laila Sara Arroyo Mühr:** Data curation; formal analysis; methodology; project administration; supervision; validation; visualization; writing – original draft. **Jiangrong Wang:** Data curation; formal analysis; investigation; methodology; validation; writing – review and editing. **Sadaf S. Hassan:** Data curation; formal analysis; investigation; methodology; writing – review and editing. **Emel Yilmaz:** Formal analysis; methodology; writing – review and editing. **Miriam K. Elfström:** Investigation; supervision; writing – review and editing. **Joakim Dillner:** Conceptualization; resources; supervision; validation; writing – review and editing.

## FUNDING INFORMATION

This study was supported by the European Union's Horizon 2020 research programme (grant No. 847845 [Risk‐stratified screening for cervical cancer, RISCC]). The funder had no role in study design, data collection and analysis, interpretation of data, decision to publish, or preparation of the manuscript.

## CONFLICT OF INTEREST STATEMENT

The authors declare no conflicts of interest.

## ETHICS STATEMENT

The study was approved by the National Ethical Review Agency of Sweden (Decision numbers 2019‐03166; 2020‐05929; and 2022‐02417‐02). In Sweden, ethical permissions are given by a national agency that is directly appointed by government, chaired by a senior judge, and has the authority to decide on requirements for consent. The project was designed in consultation with the patient association gyncancer.se. All participants gave written consent before participating in the study. Clinicaltrials.gov registration number: NCT04061967.

## Supporting information


**Data S1.** Supporting Information.

## Data Availability

Investigators interested in accessing these data for the purposes of future studies can do so by contacting the Swedish National Cervical Screening Registry (nkcx.se) after an Institutional Review Board approval has been obtained; and a Data Use Agreement being filled. Further information is available from the corresponding author upon request.
