# Supplementary material for: Nationwide registry‐based trial of risk‐stratified cervical screening
Source: Int J Cancer. 2024 Aug 15;156(2):379–88. doi: 10.1002/ijc.35142 (PMC11578075; doi:10.1002/ijc.35142)
Supplement: Supplementary file 1 — Data S1. Supporting Information. [file IJC-156-379-s001.pdf]

SUPPLEMENTARY MATERIAL

Nationwide registry-based trial of risk-stratified cervical screening

Laila Sara Arroyo Mühr, Jiangrong Wang, Sadaf S. Hassan, Emel Yilmaz, K. Miriam Elfström, and Joakim Dillner.

TABLE OF CONTENTS

Supplementary Table 1 **Page 2**

Risk estimation data sources

Supplementary Table 2 **Page 5**

Study population, participation and HPV positivity obtained in each year.

Supplementary Table 1: Risk estimation data sources

Data sources and publications used to estimate 5-year cumulative cervical cancer (CxCa) risks by risk groups according to screening findings. Most estimations are based on the Swedish national cervical screening registry (nkcx.se). When data was not available, data from the literature was used (references given below)

Risk group	Without HPV test		HPV positive	HPV16/18 genotype positive			
	Cumulative incidence proportion, % of CxCa by 5 years (source and setting)	Estimations	Cumulative incidence proportion % of CxCa by 5 years (source and setting)	Cumulative incidence proportion % of CxCa by 5 years (source and setting)	Estimations		
					Risk ratio of CxCa comparing HPV16/18 to other HPV types	Proportion of positive comparing HPV16/18 to other hrHPV types, among women positive for HPV (source and setting)	Calculation of cumulative incidence proportion for HPV16/18 positive and other HPV positive
**AGC**	2.1% (Wang et al., BMJ 2016.[1] Swedish register NKCx, cohort 1980-2011)	Not needed	9.0% (Katki et al., J Low Genit Tract Dis 2013.[4] Kaiser Permanente Northern California (KPNC) data, cotesting cohort 2006-2010)	No available data	HPV16/18 about 4 times riskier than other types	32% of HPV positive were HPV 16/18 (Stockholm screening population, NKCx 2013-2019)	Fulfill the estimated risk ratio, with the proportion of HPV16/18 genotype, splitting the cum risk of all HPV positives into cum risk of HPV16/18 positive and cum risk of other HPV types positive.
**HSIL**	2.9% (Wang et al., BMJ 2016.[1] Swedish NKCx, cohort 1980-2011)	Not needed	6.6% (Katki et al., J Low Genit Tract Dis 2013.[4] Kaiser Permanente Northern California (KPNC) data, 2ontesting cohort 2006-2010)	No available data	HPV16/18 about 3 times riskier than other types	49% of HPV positive were HPV 16/18 (Stockholm screening population, NKCx 2013-2019)	Fulfill the estimated risk ratio, with the proportion of HPV16/18 genotype, splitting the cum risk of all HPV positives into cum risk of HPV16/18 positive and cum risk of other HPV types positive.

**ASCUS/LSIL**	0.4% (Wang et al., BMJ 2016.[1] Swedish NKCx, cohort 1980-2011)	Not needed	0.5% (Katki et al., J Low Genit Tract Dis 2013.[5] Kaiser Permanente Northern California (KPNC) data, cotesting cohort 2006-2010)	No available data	HPV16/18 about 4 times riskier than other types	29% of HPV positive were HPV 16/18 (Stockholm screening population, NKCx 2013-2019)	Fulfill the estimated risk ratio, with the proportion of HPV16/18 genotype, splitting the cum risk of all HPV positives into cum risk of HPV16/18 positive and cum risk of other HPV types positive.
**HPV16/18+ve Cyto-**	Not applicable	Not applicable	Not applicable	1.3 (Follow-up of Elfström et al, PLOS Med 2021.[6] Wang J et al., Preprint.[7]	Not needed	Not needed	Not needed
**Non-attenders**	No available data	Estimated from incidence rates in Wang et al., IJC 2024.[2] (Swedish NKCx, cohort 2002-2020)	Not needed	Not needed	Not needed	Not needed	Not needed
**HPV negative**	No available data	Estimated from incidence rates in Wang et al., IJC 2024.[2] (Swedish NKCx, cohort 2002-2020)	Not needed	Not needed	Not needed	Not needed	Not needed
**Total population**	No available data	Estimated from Statistic of incidence rate of invasive CxCa in the population in 2019 (The Swedish National Cancer Registry,[3] by the Swedish National Board	Not needed	Not needed	Not needed	Not needed	Not needed

	of Health and Welfare)	

NKCx: The Swedish National Cervical Screening Registry, and its linkage with cancer registry.

References:

1. Wang J, Andrae B, Sundström K, Ström P, Ploner A, Elfström KM, et al. Risk of invasive cervical cancer after atypical glandular cells in cervical screening: nationwide cohort study. BMJ. 2016 Feb 11;352:i276.

2. Wang J, Edvardsson H, Strander B, Andrae B, Sparén P, Dillner J. Long-term follow-up of cervical cancer incidence after normal cytological findings. International Journal of Cancer. 2024;154(3):448–53.

3. Socialstyrelsen [Internet]. 2024 [cited 2024 Jul 1]. National Cancer Register. Available from: https://www.socialstyrelsen.se/en/statistics-and-data/registers/national-cancer-register/

4. Katki HA, Schiffman M, Castle PE, Fetterman B, Poitras NE, Lorey T, et al. Five-year risk of CIN3+ and cervical cancer for women with HPV-positive and HPV-negative high-grade Pap results. J Low Genit Tract Dis. 2013 Apr;17(5 0 1):S50–5.

5. Katki HA, Schiffman M, Castle PE, Fetterman B, Poitras NE, Lorey T, et al. Five-year risk of CIN3+ and cervical cancer for women with HPV testing of ASC-US Pap results. J Low Genit Tract Dis. 2013 Apr;17(5 0 1):S36–42.

6. Elfström KM, Eklund C, Lamin H, Öhman D, Hortlund M, Elfgren K, et al. Organized primary human papillomavirus–based cervical screening: A randomized healthcare policy trial. PLOS Medicine. 2021 Aug 23;18(8):e1003748.

7. Wang J, Elfström M, Dillner J. A Randomized Healthcare Policy Trial of Human Papillomavirus-Based Cervical Screening. Available at SSRN: https://ssrn.com/abstract=4845172 or http://dx.doi.org/10.2139/ssrn.4845172

Supplementary Table 2: Study population, participation and HPV positivity obtained in each year

	Recruitment	Number of women	Number of ordered	(% ordered among women)	Number of tested	(% tested among women)	Number of HPV+ve	(% HPV +ve among tested)	HPV 16	HPV 18	HPV 45	Other middle risk	Other low risk	Negative
2019	**AGC[1]**	276	54	19.57	44	15.94	6	13.64	1	2		2	1	38
	**Elderly High-grade abn[2]**	566	123	21.73	108	19.08	8	7.41	2	2			4	100
	**Elderly low-grade abn[3]**	78	14	17.95	11	14.10	4	36.36	1			2	1	7
	**Total**	920	191	20.76	163	17.72	18	11.04	4	4		4	6	145
2020	**AGC[1]**	1943	355	18.27	291	14.98	42	14.43	8	3	9	9	13	249
	**Elderly High-grade abn[2]**	3490	713	20.43	673	19.28	83	12.33	25	6	11	19	22	590
	**Elderly low-grade abn[3]**	965	214	22.18	205	21.24	29	14.15	5	0	8	11	5	176
	**Total**	6398	1282	20.04	1169	18.27	154	13.17	38	9	28	39	40	1015
2021	**AGC[1]**	382	32	8.38	19	4.97	2	10.53	1	1				17
	**Elderly[2]**	4588	951	20.73	834	18.18	104	12.47	27	6	14	22	35	730
	**HSIL[3]**	303	37	12.25	23	7.59	4	17.39	2			1	1	19
	**LSIL[4]**	159	25	15.82	16	10.06	5	31.25	2	1		2		11
	**HPV16/18 positive[5]**	1761	297	16.87	193	10.96	107	55.44	71	16	5	6	9	86
	**Non-attenders[6]**	16125	876	5.43	436	2.70	23	5.28	7	1	1	8	6	413
	**Total**	23318	2218	9.51	1521	6.52	245	16.11	110	25	20	39	51	1276

1) Women aged between 23-80, having AGC in past 0·5-6·5 years and no HPV or histopathology test in the last 2 years.

2) Women aged between 65-70 with abnormal screening findings above the age of 50, but without sufficient follow-up (risk >65/100,000).

3) Women aged 35-64, having HSIL in past 0·5-6·5 years, not having any negative HPV test afterwards, not having histology test since 2017-01-01, not having cytology test since 2018-01-01, and not having HPV positive test since 2020-01-01.

4) Women aged 35-64, having LSIL in past 0·5-6·5 years (i.e., between 2014-07-01 and 2020-06-30) and tested HPV16 or HPV18 positive, not having any negative HPV test afterwards, not having histology test since 2017-01-01, not having cytology test since 2018-01-01, and not having HPV positive test since 2020-01-01,

5) Women aged 30-80, ever having HPV16 or HPV18 positive and no cytology abnormality since 2014-07-01, not having any negative HPV test from 30 days after the positivity, not having any histology test afterwards, and not having HPV positive test since 2020-01-01 and

6) women aged 40-70, never attended screening, being invited in last 10 years, and not immigrated in last 20 years.
